# Use of pyrosequencing and DNA barcodes to monitor variations in *Firmicutes *and *Bacteroidetes *communities in the gut microbiota of obese humans

**DOI:** 10.1186/1471-2164-9-576

**Published:** 2008-12-01

**Authors:** Fabrice Armougom, Didier Raoult

**Affiliations:** 1 URMITE – UMR CNRS 6236, IRD 3R198, Université de la Méditerranée, Faculté de médecine, 27 Boulevard Jean Moulin, 13005 Marseille, France

## Abstract

**Background:**

Recent studies of 16S rRNA genes in the mammalian gut microbiota distinguished a higher *Firmicutes*/*Bacteroidetes *ratio in obese individuals compared to lean individuals. This ratio was estimated using a clonal Sanger sequencing approach which is time-consuming and requires laborious data analysis. In contrast, new high-throughput pyrosequencing technology offers an inexpensive alternative to clonal Sanger sequencing and would significantly advance our understanding of obesity via the development of a clinical diagnostic method. Here we present a cost-effective method that combines 16S rRNA pyrosequencing and DNA barcodes of the *Firmicutes *and *Bacteroidetes *16S rRNA genes to determine the *Firmicutes/Bacteroidetes *ratio in the gut microbiota of obese humans.

**Results:**

The main result was the identification of DNA barcodes targeting the *Firmicutes *and *Bacteroidetes *phyla. These barcodes were validated using previously published 16S rRNA gut microbiota clone libraries. In addition, an accurate F/B ratio was found when the DNA barcodes were applied to short pyrosequencing reads of published gut metagenomes. Finally, the barcodes were utilized to define the F/B ratio of 16S rRNA pyrosequencing data generated from brain abscess pus and cystic fibrosis sputum.

**Conclusion:**

Using DNA barcodes of *Bacteroidetes *and *Firmicutes *16S rRNA genes combined with pyrosequencing is a cost-effective method for monitoring relevant changes in the relative abundance of *Firmicutes *and *Bacteroidetes *bacterial communities in microbial ecosystems.

## Background

Investigations of the bacterial 16S rRNA genes play an essential role in the exploration of microbial diversity and bacterial taxonomy. The composition of bacterial communities is typically studied by implementing clonal Sanger sequencing of 16S rRNA PCR products [1-4]. In humans, the ratio of *Firmicutes *to *Bacteroidetes *(F/B ratio) was found to be significantly higher in obese individuals than in lean individuals. Ley and colleagues [4] demonstrated that a decrease in the F/B ratio in obese individuals correlated with weight loss over time. The authors suggested that modulation of the abundance of particular bacterial communities inherent to the gut microbiota would be beneficial for the treatment of obesity. In these experiments, the composition of the gut microbiota was monitored over a one-year period using a culture-independent method based on shotgun sequencing of 16S rRNA clone libraries. Although this method was successfully used for taxonomy characterization at the species level, it was a time-consuming and expensive [5] process that required the application of exceedingly laborious data analyses. The development of an inexpensive and quick clinical diagnostic method would significantly improve our understanding of obesity, which is a common health issue affecting a large numbers of individuals.

In this regard, the new high-throughput technology of pyrosequencing [6] offers a cost-effective alternative to traditional sequencing methods, particularly for metagenomic studies, but also for 16S rRNA-based microbial diversity studies [7-9]. A comparison of the cost and high-throughput capacity of sequencing technologies indicated that 454-Roche pyrosequencing generated far more sequence data per run at a much lower cost (30 times) than conventional dye-terminator sequencing [10]. The short length of reads (100–250 base pairs) generated by this new high-throughput technology, however, limits full length bacterial 16S rRNA sequence assembly; thus, bacterial taxonomy characterization of mixed microbial samples remains a daunting task with the risk of chimera production. In order to address this problem, recent studies have reported efficient methods for classifying short sequences [11-13] at the phylum or genus taxonomic level. In addition, short DNA-specific regions that exhibit significant variability between bacterial species have been recently investigated to avoid the computational challenge of full-length 16S rRNA sequence assembly [5,8,14,15]. Thus far, however, the sensitivity of the primers used to target short specific or variable regions over the entire bacterial domain is unclear due to the lack of exhaustive 16S rRNA sequence testing.

Since *Firmicutes *and *Bacteroidetes *are the main bacterial phyla involved in alterations of the gut microbiota in obese individuals, it would be useful to develop a faster and less expensive method for monitoring variations in their relative abundance. In this study, we report a method for rapidly estimating the ratio of *Firmicutes *to *Bacteroidetes *using thousands of pyrosequencing reads generated from near-full 16S rRNA gene amplification products or complete bacterial metagenomes. We identified two DNA barcodes as genomic signatures specific to the 16S rRNA genes of species belonging to the *Bacteroidetes *or *Firmicutes *phylum. Such DNA barcodes for bacterial phyla are not short sequence tags added to PCR products of microbial samples for massive parallel or barcoding pyrosequencing investigations [5,15].

One of the major benefits of these DNA barcodes was the ability to rapidly provide an accurate F/B ratio from a pool of thousands of short sequencing reads generated by the pyrosequencing method, without the need to assemble sequences, perform multiple sequence alignments, generate phylogenetic reconstructions, or perform BLAST analyses. Therefore, our DNA barcodes used in combination with pyrosequencing technology will be useful for clinical diagnosis and for studies involving a large spectrum of subjects and conditions such as exhaustive caloric intake and antibiotic effects.

## Results

### DNA barcodes

The *Bacteroidetes *DNA barcode is a specific sequence of 12 nucleotides, while the *Firmicutes *barcode is a degenerate sequence composed of 26 nucleotides (Table 1). Based on the 16S rRNA gene sequence from *B. fragilis *(RDP-II accession number: S000000037), the *Bacteroidetes *DNA barcode spans base pairs 537 to 548. The *Firmicutes *DNA barcode spans base pairs 1,163 to 1,188 of the 16S rRNA gene from *C. difficile *(RDP-II accession number: S000260455).

**Table 1:** Sequence and length of the identified DNA barcodes

**Phylum**	**DNA barcodes**	**Length **(bp)
***Bacteroidetes***	GGGTTTAAAGGG	12

***Firmicutes***	TCATGCCN[16]ACA	26

### Sensitivity and specificity of the DNA barcodes

**Figure 1:**
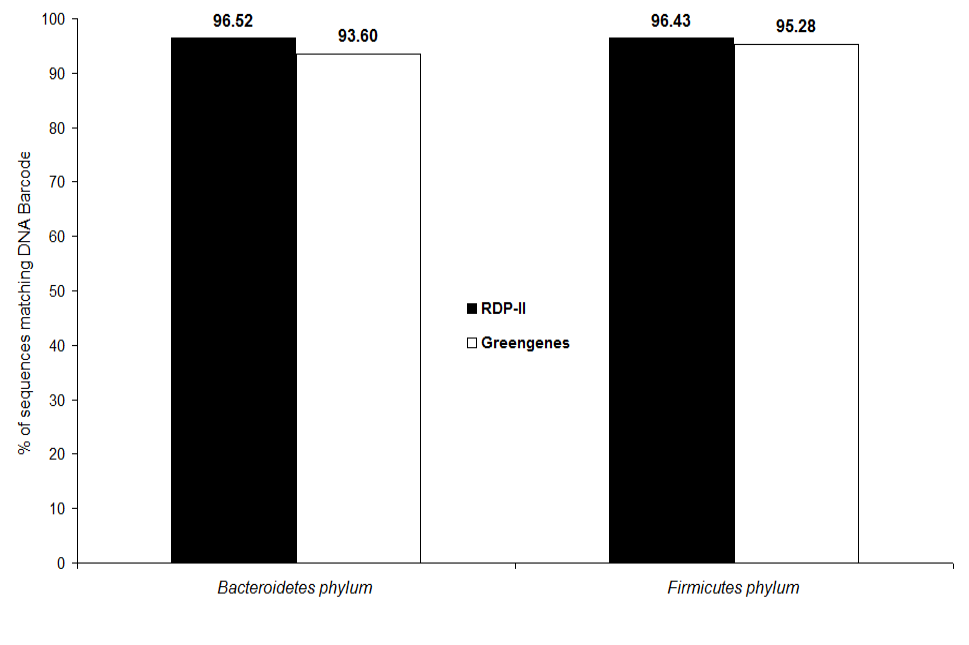
**Sensitivity of *Firmicutes *and *Bacteroidetes *DNA barcodes**. RDP-II and Greengenes are 16S rRNA databases.

Figure 1 shows the high sensitivity inherent to the use of DNA barcodes from *Bacteroidetes *and *Firmicutes *phyla when applied to the Ribosomal Database Project [16] (RDP-II) and the Greengenes [17] database. The *Bacteroidetes *DNA barcode matches 96.52% and 93.60% of the 16S rRNA sequences from the *Bacteroidetes *phylum in the RDP-II and Greengenes databases, respectively. Similarly, the *Firmicutes *DNA barcode matches 96.43% and 95.28% of the 16S rRNA sequences from the *Firmicutes *phylum in the RDP-II and Greengenes databases, respectively.

The *Bacteroidetes *DNA barcode is also highly specific given that 99.98% of the 113,000 16S rRNA sequences from the RDP-II database do not possess this DNA barcode (Table 2). There are actually only 18 *Firmicutes *sequences, two *Planctomycetes *sequences, and one *Cyanobacteria *sequence that possess the *Bacteroidetes *DNA barcode. Using the Greengenes database, the *Bacteroidetes *DNA barcode was less specific, demonstrating sequence matches with a greater number of phyla (7 of 15). The *Firmicutes *DNA barcode was also highly specific, with 98.25% of all tested 16S rRNA sequences from the RDP-II database lacking this DNA barcode. Compared to the *Bacteroidetes *DNA barcode, the loss in specificity observed with the *Firmicutes *DNA barcode is due to the fact that the latter matches 97.38% and 32.76% of sequences belonging to the *Fusobacteria *and *Cyanobacteria *phyla, respectively, using the RDP-II database (Table 2). However, These two phyla are poorly represented in the intestinal microbial flora (< 0.15% of the total 16S rRNA sequences in each study) [1-4]. Finally, using the 335,830 sequences available in the RDP-II database, the false-discovery rates for the *Firmicutes *and the *Bacteroidetes *DNA barcode were found to be very low, 0.90% (3035 of 335830) and 0.06% (105 of 335830), respectively.

**Table 2:** Specificity of the *Firmicutes *and *Bacteroidetes *DNA barcodes

			**% of Hit with *Bacteroidetes *barcode**		**% of Hit with *Firmicutes *barcode**	
**Phylum**	**Number of sequences in RDP-II**	**Number of sequences in Greengenes**	**RDP-II**	**Greengenes**	**RDP-II**	**Greengenes**

*Bacteroidetes*	18272	4718	_	_	0.41	0

*Firmicutes*	45757	16023	0.04	0.057	_	_

*Proteobacteria*	41277	38449	0	0.049	0.18	0.27

*Actinobacteria*	11366	12736	0	0.0079	1.28	1.07

*Cyanobacteria*	2164	1873	0.05	0.053	32.76	29.31

*Spirochaetes*	1547	1677	0	0.059	0	0.18

*Verrucomicrobia*	953	324	0	0	0	0

*Planctomycetes*	864	553	0.23	0.54	0	0.18

*Chloroflexi*	713	643	0	0	0	0.77

*Acidobacteria*	782	865	0	0	0	0

*Aquificae*	739	1131	0	0	0	0

*Fusobacteria*	421	198	0	0	97.38	90.40

*Nitrospirae*	376	156	0	0	0	0

*Deinococcus-Thermus*	381	419	0	0.23	0	0.24

*Chlamydiae*	178	167	0	0	0	0

*Deferribacteres*	71	64	0	0	0	0

RDP-II: Ribosomal Database Project II. For each bacterial phylum of the RDP-II and Greengenes databases, the percentage of sequences possessing the *Bacteroidetes *and/or the *Firmicutes *DNA barcode was calculated.

### Validation of DNA barcodes using full 16S rRNA gene sequences

The 16S rRNA surveys based on the shotgun sequencing method from 16S rRNA clone libraries allow full 16S rRNA sequence assembly and can determine the relative abundance of a bacterial phylum using the phylogeny reconstruction ARB tool [18]. No significant statistical difference (P > 0.05) was found when the DNA barcode method was used to assess *Bacteroidetes *and *Firmicutes *communities using four published 16S rRNA surveys of the gut microbiota (Table 3).

**Table 3:** Assessment of communities' abundance using clonal sequencing data

**Origin of data**	**Bacterial phylum**	**Bacterial proportion (%) found in the study**	**Bacterial proportion (%) using DNA barcode**
Eckburg *et al. *study [1]	*Firmicutes*	50.78	50.36
	
	*Bacteroidetes*	47.67	47.52

Ley *et al. *mouse gut study [3]	*Bacteroidetes *(+/+ Lean)	36.39	36.45
	
	*Bacteroidetes *(Ob/ob Obese)	22.86	22.27

Turnbaugh *et al. *study [2]	*Firmicutes *(Ob/ob Donors)	67.86	62.78
	
	*Bacteroidetes *(Ob/ob Donors)	28.74	29.04
	
	*Firmicutes *(Ob/ob recipients)	62.83	60.32
	
	*Bacteroidetes *(Ob/ob recipients)	31.88	31.27
	
	*Firmicutes *(Lean donors)	54.82	54.82
	
	*Bacteroidetes *(Lean donors)	40.96	40.96
	
	*Firmicutes *(Lean recipients)	49.39	48.26
	
	*Bacteroidetes *(Lean recipients)	47.7	46.69

Ley *et al. *human gut study [4]	*Firmicutes *(0 week on diet)	88.47	84.97
	
	*Bacteroidetes *(0 week on diet)	3.15	3.11
	
	*Firmicutes *(12 week on)	85.35	82.16
	
	*Bacteroidetes *(12 week on diet)	9.58	9.36
	
	*Firmicutes *(26 week on diet)	70.91	66.97
	
	*Bacteroidetes *(26 week on diet)	12.92	12.8
	
	*Firmicutes *(52 week on diet)	75.3	70.66
	
	*Bacteroidetes *(52 week on diet)	15.66	15.02

Bacterial relative abundances found in four 16S rRNA surveys were compared to those inferred with the DNA barcodes application.

The first dataset (11,831 sequences) was obtained from a study of the diversity of human intestinal microbial flora [1] that demonstrated the *Firmicutes *and *Bacteroidetes *phyla represent 50.78% and 47.67%, respectively, of all 16S rRNA sequences. Applying our DNA barcode method to the same dataset, we observed that 50.36% and 47.52% of this dataset were represented by the *Firmicutes *and the *Bacteroidetes *phyla, respectively (Table 3). Thus, the percentages obtained for the two bacterial phyla using the DNA barcodes or ARB tool were very close. A second dataset was obtained from a study of the gut microbiota of obese mice [3] and included 5,088 16S rRNA sequences. In this study, Ley *et al. *reported a 50% reduction in the abundance of *Bacteroidetes *within obese mice compared to a control group of lean mice. For the subset of mice with the obese genotype, our estimate of the relative abundance of *Bacteroidetes *was 22.27%, while the authors reported a relative abundance of 22.86%. For the subset of mice in the control group (lean mice), our method provided a *Bacteroidetes *abundance estimate of 36.45%, while the authors found a relative abundance of 36.39% (Table 3).

In another study, Turnbaugh and colleagues analyzed the increased capacity of the microbiome of obese mice to harvest energy from their diet [2]. From a dataset consisting of 4,157 16S rRNA sequences, the authors demonstrated that the relative abundance of *Bacteroidetes *in the obese donor and obese recipient groups was 28.74% and 31.88%, respectively. For these two groups, our results indicated a nearly identical abundance of *Bacteroidetes *(29.04% and 31.27%, respectively). In addition, the relative abundance of *Bacteroidetes *in the lean groups was equivalent using these two methods. The relative abundance of *Firmicutes *for the two obese groups, however, was slightly different using our method (62.78% and 60.32%, respectively) and that reported by Turnbaugh *et al*. (67.86% and 62.83%, respectively) (Table 3).

The final dataset was retrieved from a survey of 16S rRNA sequences found in the gut microbiota of obese humans subjected to a specific calorie-diet [4] and monitored for the relative abundance of *Bacteroidetes *and *Firmicutes*. While the differences between our results and those of this study did not exceed 0.54% with regard to estimation of *Bacteroidetes *abundance, the relative abundance of *Firmicutes *observed using these two methods differed by up to 4.64% (Table 3).

### Discrepancies

**Figure 2:**
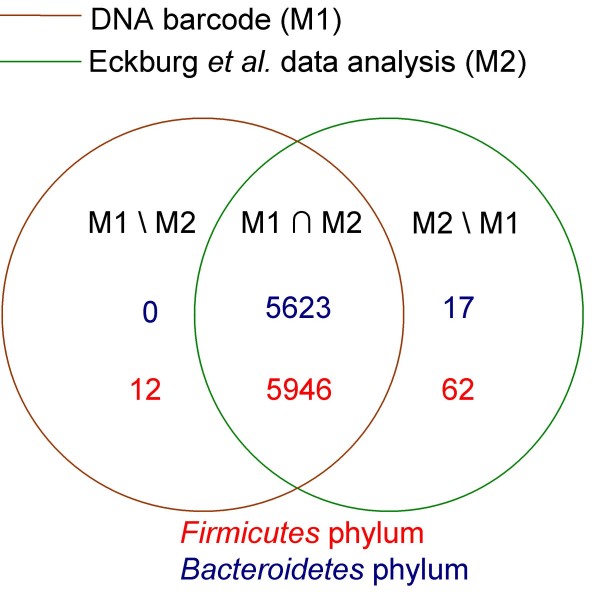
**Discrepancies in sequence repartition of bacterial communities**. M1 ∩ M2: in M1 and M2; M1\M2: in M1 out of M2; M2\M1: in M2 out of M1. The blue and red figures are the number of sequences, respectively classified in *Bacteroidetes*, *Firmicutes *phyla by M1 or M2, or by both of them.

There were only a few 16S rRNA sequences that were resistant to equivalent taxonomic classification using the three analytical methods: the ARB tool, the DNA barcode method, and the RDP-II classifier. The vast majority of 16S rRNA sequences assigned to either the *Firmicutes *or *Bacteroidetes *phyla were similarly classified using the ARB tool (M1) or our DNA barcode (M2), therefore representing the "core" 16S rRNA sequence assignments (Figure 2, M1 n M2). In addition to this core, assignment of a small number of 16S rRNA sequences to the *Firmicutes *and *Bacteroidetes *phyla was performed exclusively by one method or the other (Figure 2 M1\M2 and M2\M1), and thus accounting for the discrepancies or ambiguous taxonomic assignments.

**Figure 3:**
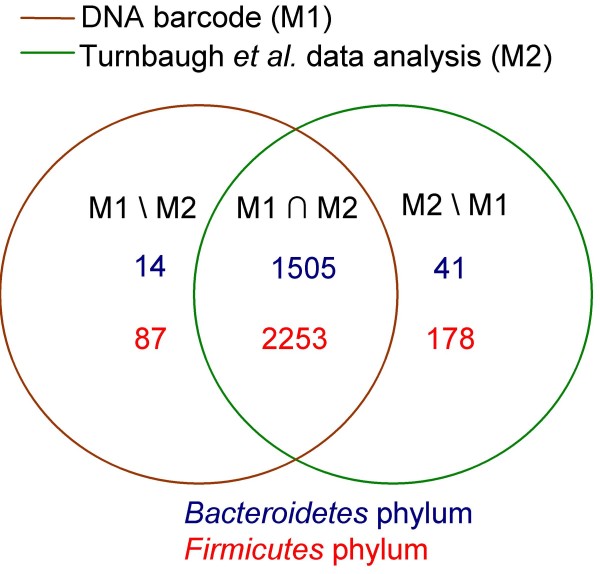
**Discrepancies in sequence repartition of bacterial communities**. M1 ∩ M2: In M1 and M2; M1\M2: in M1 out of M2; M2\M1: in M2 out of M1. The blue and red figures are the number of sequences, respectively classified in *Bacteroidetes *and *Firmicutes *phyla by M1 or M2, or by both of them.

With regard to the 16S rRNA data presented by Eckburg *et al. *[1], 17 of these sequences were assigned to the (*Bacteroidetes*) M2\M1 group, 62 were assigned to the (Firmicutes) M2\M1 group, and 12 were assigned to the (*Firmicutes*) M1\M2 group (Figure 2). The most significant discrepancy between M1 and M2 was observed with the 16S rRNA data from Turnbaugh *et al. *[2]. In this report, 178 sequences were assigned to the (*Firmicutes*) M2\M1 group, 88 were assigned to the (*Firmicutes*) M1\M2 group, 14 were assigned to the (*Bacteroidetes*) M1\M2 group, and 41 were assigned to the (*Bacteroidetes*) M2\M1 group (Figure 3).

The "RDP-II Classifier" (RC) method was used to obtain additional 16S rRNA taxonomic assignments for the sequences defined as discrepancies between M1 and M2. We evaluated the agreement between the RPD-II classifier and the sequence assignments of M1 and M2.

The 12 sequences in the (*Firmicutes*) M1\M2 group (Figure 2), which were assigned to the *Firmicutes *phylum by our DNA barcode method, were classified into the *Fusobacteria *phylum (nine sequences) and into a group related to the *Cyanobacteria *phylum (three sequences) in the Eckburg *et al. *study. Similarly, analyses using the RC method resulted in the same classification as Eckburg's analysis with a bootstrap confidence (Bc) superior to 95%, suggesting that our *Firmicutes *DNA barcodes provided 12 false-positive results. This result was not surprising given that our *Firmicutes *barcode matched a significant proportion of sequences belonging to the *Fusobacteria *and *Cyanobacteria *phyla in the RDP-II and Greengenes databases (Table 2). Moreover, the 62 sequences assigned to the (*Firmicutes*) M2\M1 group and the 17 sequences assigned to the (*Bacteroidetes*) M2\M1 group were similarly classified using the RC method, confirming the classification reported by Eckburg *et al. *and demonstrating that our DNA barcode method failed only in the appropriate taxonomic assignment of a small number of sequences.

Based on the16S rRNA data obtained by Turnbaugh *et al.*, seven sequences among the 41 sequences assigned to the (*Bacteroidetes*) M2\M1 group were not assigned by RC to the *Bacteroidetes *phylum as expected, but rather to the *Firmicutes *phylum (Bc > 90%). Interestingly, these seven sequences also possessed the *Firmicutes *DNA barcode. In addition, of the 14 sequences assigned to the *(Bacteroidetes*) M1\M2 group, six sequences were also classified as *Bacteroidetes *using the RC method (Bc > 90%). Likewise, from the 178 sequences assigned to the (*Firmicutes*) M2\M1 group, the RC method classified five sequences in the *Bacteroidetes *phylum (Bc > 90%) and not in the *Firmicutes *phylum. Moreover, of the 88 sequences assigned to the (*Firmicutes*) M1\M2 group, only four were classified as *Firmicutes *(Bc > 90%) using RC, suggesting the possibility of 84 false-positives as a consequence of DNA barcode application.

Thus, regardless of the method used, these results demonstrate the difficulty inherent to establishing the correct phylum classification for a small number of complete 16S rRNA sequences.

### DNA barcodes applied to short 16S rRNA pyrosequencing reads

Two pyrosequencing libraries generated in our laboratory for studies related to bacterial diversity in mixed microbial samples were analyzed. The first library was obtained from a brain abscess of a patient at a Marseille hospital and contained 2,612 reads with an average length of 95.78 base pairs. In parallel, results using classical Sanger sequencing of a 16S rRNA clone library from the same sample were used as a source of comparison. We analyzed the F/B ratios using two different culture-independent methods: 16S rRNA pyrosequencing and 16S rRNA clonal Sanger sequencing. The results of the 16S rRNA clonal Sanger sequencing method indicated that 50 of the clone sequences belonged to the *Bacteroidetes *phylum, while 49 belonged to the *Firmicutes *phylum, resulting in an F/B ratio of 0.98 (Table 4). Application of the DNA barcode method indicated that 41 of the pyrosequencing reads belonged to the *Bacteroidetes *phylum, while 39 belonged to the *Firmicutes *phylum, resulting in an F/B ratio of 0.95. We also analyzed the 2,612 reads using the sequence classification tool RDP-II classifier. The F/B ratio obtained using this tool was 1.02 (Table 4); however, 1.03% of all pyrosequencing reads obtained were too short in length to be classified by the RDP-II tool. In addition and contrary to our DNA barcode classification, the RDP-II classifier limits the number of sequences that can be submitted (30,000 per run). Finally, compared to the F/B ratio obtained with the RDP-II classifier tool (1.02), the ratio obtained with our DNA barcode method (0.95) was closer to that obtained by clonal Sanger sequencing (0.98).

**Table 4:** Determination of F/B ratios using pyrosequencing and clonal sequencing

**Sample**	**Sequencing method**	**Estimation method**	***F***	***B***	**ratio F/B**
Brain abscess pus	16S rRNA P	Barcode hits	39	41	0.95
	
	16S rRNA P	RDP-II classifier	521	509	1.02
	
	16S rRNA CS	Number of clone sequences	49	50	0.98

Cystic fibrosis sputum	16S rRNA P	Barcode hits	34	2	17
	
	16S rRNA CS	Number of clone sequences	16	1	16

Caecal content	MP	Barcode hits	79	33	2.39
		
		% of total 16S rRNA sequences	60.70	28.41	2.14

P, CS and MP mean Pyrosequencing, Clonal Sanger Sequencing and Metagenome Pyrosequencing, respectively. *F *and *B *mean *Firmicutes *phylum and *Bacteroidetes *phylum, respectively. The numbers of clone sequences assigned to the *Firmicutes *or *Bacteroidetes *phylum was performed using BLAST algorithm and Genebank database. For the metagenome pyrosequencing, the 16S rRNA fraction was found with BLAST algorithm and RDP-II database. RDP-II classifier was used as a control with a bootstrap confidence > 80%

The pyrosequencing error rate in the *Bacteroidetes *barcode sequence, which contains 4 homopolymers (GGGTTTAAAGGG), was estimated to 8.89% (4 errors/45) for this dataset. In all cases, the sequencing errors in the *Bacteroidetes *barcode sequence were due to a one base insertion (A) in the homopolymer A. One of these cases also showed a one base insertion (T) in the homopolymer T (Table 5).

**Table 5:** Pyrosequencing errors

**Read label**	**Pyrosequencing errors in *Bacteroidetes *barcode**
002964_0126_2423	GGGTTT**AAAA**GGG
000955_0116_2845	GGGTTT**AAAA**GGG
003177_0112_2481	GGGTTT**AAAA**GGG
001840_0197_1892	GGG**TTTTAAAA**GGG

Sequencing errors found in the *Bacteroidetes *barcode 'GGGTTTAAAGG' using the brain abscess pyrosequencing dataset. The sequencing errors were identified as an A (4/4) and a T (1/4) base insertion.

A second pyrosequencing library from the sputum of a cystic fibrosis (CF) patient from a Marseille hospital was also analyzed. This library contained 4,499 reads with an average length of 93.05 base pairs. A 16S rRNA clonal Sanger sequencing approach was also applied to the same CF sputum. The results of the two culture-independent methods were similar, as indicated in Table 4. Results from the clonal Sanger sequencing analysis indicated that 16 clone sequences belonged to the *Firmicutes *phylum, while only one sequence was assigned to the *Bacteroidetes *phylum (F/B = 16). Analysis of the 16S rRNA pyrosequencing data indicated that the *Firmicutes *DNA barcode matched 34 sequence reads, while the *Bacteroidetes *DNA barcode matched two sequence reads (F/B = 17).

### DNA barcodes applied to metagenome pyrosequencing

From the metagenomic study performed by Turnbaugh *et al.*[2], 1,046,611 and 677,384 pyrosequencing reads were collected from Lean1 and Obese1 mice, respectively, using the GS 20 pyrosequencer. The authors analyzed the merged data (1,723,995 reads) by BLASTing against the 16S rRNA RDP-II database. Using this method, the authors determined the total number of sequence reads classified as 16S rRNA genes and the number of sequence reads belonging to *Bacteroidetes *and *Firmicutes *phyla. This analysis led to the determination of an F/B ratio of 2.14. Using our DNA barcode method with this dataset assigned 79 reads to the *Firmicutes *phylum and 33 reads to the *Bacteroidetes *phylum, resulting in an F/B ratio of 2.39 (Table 4).

## Discussion

Owing to the rapid accumulation of data coupled with advances in sequencing technology – including the 454 Life Sciences GS FLX System sequencer [6], which generates more than 100 million bases per run – the development of methods capable of rapidly processing these data has become essential. Thus, the aim of the present investigation was to provide a straightforward, accurate, inexpensive and rapid tool to estimate the relative abundance of bacterial communities and the resulting F/B ratios from thousands of 16S rRNA short sequencing reads, without the need for any assembly procedure, multiple sequence alignment, BLAST analysis, or phylogeny reconstruction.

The DNA barcode obtained for the *Bacteroidetes *phylum is sensitive and specific. In the literature, Dick and Field reported a 16S rRNA *Bacteroidetes *barcode that can be used as a probe to estimate the occurrence of fecal *Bacteroidetes *[19]. As observed with our DNA barcode using the RDP-II database, assessment of the sensitivity and specificity of the *Bacteroidetes *DNA barcode reported by Dick and Field produced a sensitivity score of 88.00% (compared to 96.52% for our *Bacteroidetes *barcode) and a specificity score of a 99.25% (compared to 99.98% for our *Bacteroidetes *barcode). Thus, our *Bacteroidetes *barcode possesses both greater sensitivity and greater specificity. Although the *Fusobacteria *and *Cyanobacteria *phyla are responsible for a slight decrease in the specificity of our *Firmicutes *barcode, the relative proportion of both of these phyla is marginal (less than 0.15% of all 16S rRNA sequences in each 16S rRNA survey [1-4]).

Compared to the nearly completed 16S rRNA sequences assembled from four clone library surveys of gut microbiota, the results obtained using our 16S rRNA barcode application are in agreement (P > 0.05). Importantly, there are some discrepancies that highlight several limitations of the bioinformatics and biological methodologies. First, it is possible that some sequences belonging to the *Firmicutes *or *Bacteroidetes *phyla may not be identified using our method since the sensitivity of our DNA barcode is less than 100%. Moreover, due to limited specificity (less than 100% for our DNA barcode), some false-positives may be introduced into the results. Another discrepancy is caused by the requirement for polymerase chain reaction and its capacity to generate sequencing errors. A single base sequencing error (substitution or deletion) located in the region of a conserved nucleotide comprising the DNA barcode may result in a false-positive or false-negative result. The quality of the sequencing analysis and the sequence assembly process are important to the quality of the results obtained using the DNA barcode method.

The results provided by the RDP-II "classifier" taxonomic assignment method suggest that there are few errors in the taxonomic classification of the 16S rRNA sequences from the clone libraries and emphasize the need to benchmark the tools used in such analyses. To estimate the abundance of phyla within a bacterial community using the phylogenetic reconstruction ARB tool, 16S rRNA surveys of the intestinal microbial flora first target the computation of a multiple sequence alignment (MSA) with the NAST multialigner [20] or the autoaligner of the ARB software. It is obvious that the quality of the phylogenetic reconstruction is directly related to the accuracy of the sequence alignment analysis. In many cases, an alignment is considered biologically satisfactory when it accurately reflects the structural relationship between the given sequences. As a consequence, MSA algorithms are typically benchmarked with a collection of structure-based sequence alignments [21,22], which are considered to be gold standards [23]. In contrast, the processing methods involved in 16S rRNA studies suffer from a lack of benchmarking tests or gold standard references [24]. Thus, the recent MSA programs [18,20] capable of aligning large quantities of 16S rRNA sequences must be evaluated for their alignment accuracy, as currently performed for Muscle, T-COFFEE, MAFFT, Probcons, and the Clustalw MSA programs for protein sequences [25,26]. Recently, Carroll *et al. *[27] proposed the first DNA database of 3,545 DNA reference alignments. Finally, because the accuracy of phylogeny reconstruction depends on the number of informative sites, the short pyrosequencing reads collected by the GS 20 are theoretically not suitable for inferring phylogenic reconstruction. In a study performed by Zongzhi *et al. *[14], however, alignment of short pyrosequencing reads by NAST and insertion of these sequences into a pre-established phylogenic tree of full-length 16S rRNA gene sequences using ARB provided satisfactory results. While laborious data analyses involving sequencing of the 16S rRNA clone library enable the characterization of bacterial taxonomy at the species level, they do not represent an effective, low-cost strategy for clinical diagnosis or for monitoring F/B ratio variations in a large spectrum of obese humans.

In contrast, 16S rRNA or metagenome pyrosequencing offers an inexpensive and rapid strategy that can exploit the use of a DNA barcode representative of a bacterial phylum to process thousands of short sequence reads. The short read lengths of pyrosequencing are sufficient to successfully estimate F/B ratios using the DNA barcode method, but because our *Bacteroidetes *barcode contains homopolymers and because errors in pyrosequencing reads (indels and ambiguous bases) occur most often in homopolymeric regions [28], the proportion of *Bacteroidetes *can be underestimated (Table 5). Finally, we assume a similar pyrosequencing error rate in the *Bacteroidetes *and in the *Firmicutes *barcodes since the F/B ratios obtained with the pyrosequencing and the clonal Sanger sequencing data were closed (Table 4).

A short pattern search against a significantly large sequence database (e.g., metagenome data) is less time-consuming with respect to CPU time and much faster than performing the BLAST search algorithm against a 16S rRNA database. Moreover, results from the BLAST analysis require that the identified species be grouped by phylum using a tool such as the ARB tool.

The evolution of pyrosequencing strategies has focused on targeting of specific regions [14] and maximizing multiplexing capabilities (massive parallel or barcoding pyrosequencing), allowing independent samples to be pooled together and sequenced in a single run due to a short tag carried at the 5'end of the primer [5,15]. Since the DNA barcodes for *Firmicutes *and *Bacteroidetes *are separated by approximately 600 bp, they cannot be sequenced in the same read using pyrosequencing technology. An alternative method using distinct DNA amplification (with two primer sets) of the 16S rRNA region of *Bacteroidetes *and *Firmicutes *barcodes would not be an effective strategy due to differences in amplification efficiency [29]. In the near future, however, rapid technical advances (~400–500 bases for the next-generation GS FLX titanium instrument) will likely increase the read length and overcome this drawback, which currently prevents massive parallel and specific region pyrosequencing strategies.

Finally, these DNA barcodes should permit the development of real-time PCR assays using the barcode as a probe. This will be an elegant, low cost, and effective application for day-to-day use in clinical settings.

## Conclusion

Based on a DNA barcode for 16S rRNA gene sequences, we have proposed a useful and practical, yet low cost strategy to effectively evaluate obesity in humans. This is accomplished using a method that rapidly determines the F/B ratio present in a patient. Our DNA barcodes target the two major phyla of the gastrointestinal bacterial community, which show changes in their abundance in obese humans. Additionally, these DNA barcodes are capable of rapidly processing thousands of short sequencing reads. The F/B ratios that result from 16S rRNA clonal Sanger sequencing, 16S rRNA pyrosequencing, and metagenome pyrosequencing can be accurately estimated using our DNA barcodes.

The short length of the reads that result from high-throughput pyrosequencing coupled with the high degree of nucleotide conservation among the 16S rRNA genes prevents sequence assembly. Consequently, short DNA fragments exploited as DNA tags or barcodes that enable the characterization of taxonomy at the phylum, genus, or species level represent tools that are adapted to assist in clinical diagnosis and monitoring relevant changes in the relative abundance of bacterial communities in microbial ecosystems.

## Methods

### 16S rRNA RDP-II and Greengenes databases

The 16S rRNA sequences of the bacterial phyla were downloaded from the Ribosomal Database Project-II site [30]. Both "isolates" and "uncultured" sequences greater than 1,200 bp were selected. Only those sequences defined as "Good Quality" were retrieved. From the Greengenes database [31], the sequences of the bacterial phyla were exported using the NCBI taxonomy.

### Full 16S rRNA sequences retrieved from clonal Sanger sequencing datasets

The 16S rRNA datasets (sequences, alignment, and phylogenetic tree) obtained from the Ley *et al. *16S rRNA surveys [3,4] were retrieved from an ARB file located at [32] and [33]. For each ARB file, the sequences assigned to *Firmicutes *and *Bacteroidetes *were extracted and imported in Fasta file format. The relative abundance of the major bacterial communities of all complete 16S rRNA gene studies was calculated from the Fasta format files. It was necessary to install the ARB software package [34] to access these data. The 16S rRNA dataset (sequences, alignment, and phylogenetic tree) obtained from the Eckburg *et al. *study [1] were downloaded from [35]. Complementary results were obtained upon request. The last 16S rRNA dataset (sequences, alignment, and phylogenetic tree) acquired from the Turnbaugh *et al. *study [2] was selected from [36].

### DNA barcode identification

**Figure 4:**
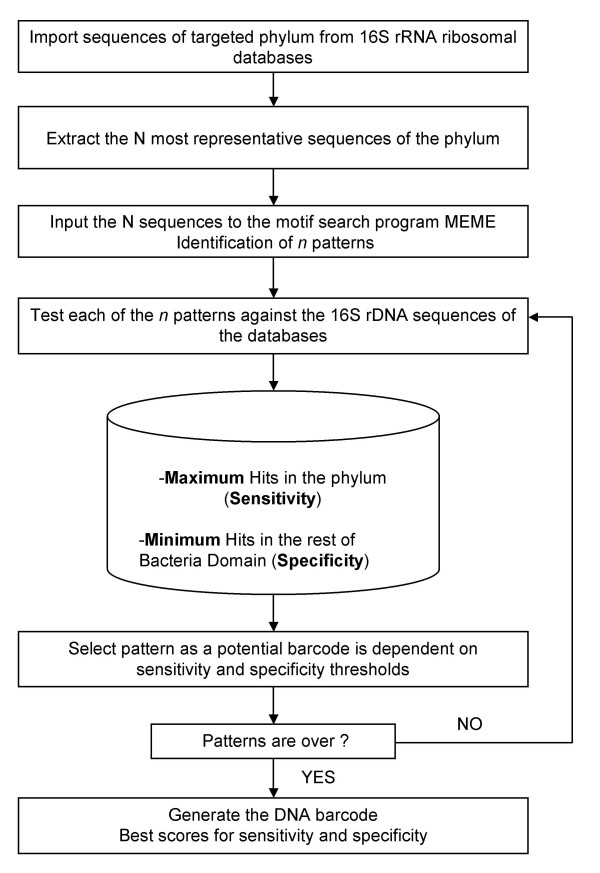
Flow chart summarizing the procedure for identifying the DNA barcodes.

The general procedure used to define the final DNA barcode is described in the flow chart in Figure 4. Extraction of the N most representative sequences was performed with the Seq_reformat program, a part of the T-COFFEE package [37]. This extraction discarded sequence redundancy and reduced the time calculation for pattern search. The MEME search motif program [38] was installed locally and run using default options. The patterns were tested against the 16S rRNA sequences of the databases using Dreg, an EMBOSS package program. For *Firmicutes*, the final barcode was refined manually via multiple sequence alignments of the phylum. Muscle version 3.56 was performed for the multiple sequence alignment (MSA) and Seaview [39] executable for the MSA edition.

### Evaluation of DNA barcodes with reduced 16S rRNA data

The *Firmicutes *signature was not applied to the dataset obtained from the Ley *et al. *study [3] because more than 1,500 sequences of the 5,088 had a length inferior to 1,200 bp. Similarly, the dataset obtained from the Ley *et al. *study [4] was reduced from 18,348 to 16,615 sequences because several sequences were too short to potentially possess the *Firmicutes *barcode.

### Sensitivity and specificity of DNA barcodes

The sensitivity of the DNA barcode was defined as the fraction of 16S rRNA sequences of the phylum that possessed the barcode. The specificity of the DNA barcode was defined as the fraction of sequences of a phylum that did not possess the barcode.

### Taxonomic assignment

We used a Naïve Bayesian rRNA classifier [40] to compare discrepancies between the results of our DNA barcode method and those of 16S rRNA surveys [1-4].

### Discrepancies

M1 represents the DNA barcode method used herein, and M2 is the method applied in the 16S rRNA studies [1-4]. M1 n M2 was the number of sequences identically classified by both methods. The *n*(*Firmicutes*) M1\M2 indicates *n *sequences were assigned to *Firmicutes *phylum by M1, but not by M2. The *n*(Bacteroidetes) M2\M1 indicates *n *sequences were assigned to the *Bacteroidetes *phylum by M2, but not by M1.

### Clonal Sanger sequencing libraries

The DNA extraction, genomic amplification, cloning procedures, and sequencing are described in Bittar *et al. *[41]. From the brain abscess cerebral sample, a library of 100 sequencing clones was analyzed. From the cystic fibrosis sputum, a library of 36 sequencing clones was analyzed.

### Sequencing errors in the Bacteroidetes barcode

Identification of sequencing errors in the *Bacteroidetes *barcode was performed by BLAST similarity search against the pyrosequencing dataset of the brain abscess sample using an extended barcode consensus sequence (CCGGANTTATTGGGTTTAAAGGGNGCG) from all the *Bacteroidetes *sequences identified by clonal Sanger sequencing. Reads classified as member of the *Bacteroidetes *phylum by RDP-II classifier (BP > 95) and with sequencing errors in the *Bacteroidetes *barcode were labelled 002964_0126_2423, 000955_0116_2845, 003177_0112_2481 and 001840_0197_1892.

### Pyrosequencing libraries

The 16S pyrosequencing sequences from cystic fibrosis sputum were deposited in the NCBI Short Read Archive under accession number SRS001099 and taxid 433733. The 16S pyrosequencing sequences from the pus of a brain abscess were deposited in the NCBI Short Read Archive under accession number SRS001098 and taxid 539654. Following the conditions detailed in Margulies *et al. *[6], PCR products (the amplicon size is about 1460 bp) were sequenced with the GS 20 platform (454 Life Science-Roche) using a titration 40 × 75 Picotitreplate™ (PTP) with eight regions. Four conditions were tested and duplicated as described by the Roche procedure, and bioinformatics analysis was performed for the dataset from the region that was closest to the optimal condition (accession number SRS001099 and SRS001098) of one DNA copy per bead.

## Competing interests

The authors declare that they have no competing interests.

## Authors' contributions

FA designed and performed all data analyses and wrote the first draft of the manuscript. DR conceived of the study and helped draft the manuscript. Both authors read and approved the final version of the manuscript.

## Funding

This work was funded by the Network of Excellence European Pathogenomics.
